# Sodium fluoride PET imaging as a quantitative pharmacodynamic biomarker for bone homeostasis during anti-DKK1 therapy for multiple myeloma

**DOI:** 10.1038/bcj.2017.95

**Published:** 2017-10-06

**Authors:** Y Wang, A J Yee, C Sirard, S Landau, N Raje, U Mahmood

**Affiliations:** 1 Department of Radiology, Massachusetts General Hospital, Boston, MA, USA; 2 Harvard Medical School, Boston, MA, USA; 3 Center for Multiple Myeloma, Massachusetts General Hospital Cancer Center, Boston, MA, USA; 4 Leap Therapeutics, Inc., Cambridge, MA, USA

Multiple myeloma (MM) is a malignancy of plasma cells that characteristically localizes to bone, resulting in osteolytic lesions in 80% of patients.^1^ Patients responding to standard therapies have lower levels of serum DKK1, a negative regulator of bone formation, suggesting that myeloma cells are the main source of circulating DKK1 protein.^2^ As such, DKK1 is a promising target for treatment of MM-associated bone disease.^3^ A phase IB clinical study of BHQ880, a humanized IgG4 monoclonal antibody with neutralizing activity against DKK1, has already shown a general trend toward increased bone mineral density (BMD) over time in treated subjects.^4^

Fluoride ion (F−) is a bone-seeking agent with applications for imaging metabolic, traumatic, malignant and inflammatory bone diseases.^5^ 18F-NaF positron emission tomography (PET) has been demonstrated to be precise and reproducible for the quantitative measure of global bone metabolism.^6, 7^ Similar to fluorodeoxyglucose PET, NaF PET imaging allows dynamic imaging with quantitation of kinetics of fluoride accumulation and deposition.^8^ We hypothesized that static and dynamic NaF PET imaging could detect changes in local bone homeostasis and be used to monitor changes in response to MM therapies that directly and indirectly alter DKK1 levels.

To investigate our hypothesis, we enrolled eight consecutive patients from a phase I clinical trial of DKN-01, a humanized monoclonal antibody targeting DKK1 (Clinicaltrials.gov NCT01711671). DKN-01 was given 300 mg intravenously on days 1 and 15 on a 28-day cycle, versus lenalidomide and dexamethasone (standard of care). The clinical study received Institutional Review Board approval. Informed consent was obtained from all patients. Patient demographics are summarized in Table 1. The primary inclusion criteria were age ⩾30 years, history of relapsed or refractory MM treated with at least 1 prior line of treatment, measurable disease, osteolytic bone lesions, an Eastern Cooperative Oncology Group scale performance of 0 or 1, an estimated life of at least 26 weeks and adequate organ function. All patients had prior bisphosphonate treatment, with a median of 65 days (range 12–273 days) since last treatment before enrolling on the trial.

Six patients underwent (1) static NaF PET/computed tomography (CT) imaging, three with and three without dynamic imaging of the thorax, and (2) dual-energy X-ray absorptiometry (DEXA) before study entry and after completing six cycles of DKN-01 therapy. An additional patient underwent baseline and cycle 3 imaging, as DKN-01 was discontinued after three cycles due to an adverse event (rash). Mean interval between post-treatment PET/CT imaging and therapy completion was 25 days (range 21–28). One patient received lenalidomide and dexamethasone without DKN-01 and underwent baseline imaging only.

Among the seven patients treated with DKN-01 and standard of care, the treatment was tolerated well. There was only one grade 3 event, rash, related to lenalidomide. The overall response rate by International Myeloma Working Group criteria^9^ was 57.1% (4/7), with one patient achieving a best response of very good partial response and three patients experiencing a best response of partial response. The median time to best response was 9.3 months.

Mean standardized uptake values (SUVmean) on static NaF PET/CT were measured by drawing a volume of interest (VOI) encircling the anatomy of interest using a multimodality workstation (Seimens TrueD, Erlangen, Germany). The boundaries of the VOI were defined by the co-registered noncontrast CT; the lower threshold value was set at 450 Hounsfield unit to exclude non-cortical bone; upper threshold value defaulted to the HUmax within the VOI as a feature of the analysis software.

SUVmean at L4 was 5 (range 4–6.9) at baseline and 5.6 (range 4.4–7.2) at cycle 6. Mean percentage change in SUVmean between baseline and cycle 6 was 10.2% (range 0–29%), a statistically significant increase (*P*=0.036 by paired *t*-test). Change in cortical SUVmean at L4 in the patient with cycle 3 imaging was 2% compared to baseline.

SUVmean at the left hip was 2.9 (range 1.5 to 3.3) at baseline and 3.1 (range 1.5 to 4.1) at cycle 6. Mean percentage change in SUVmean between baseline and cycle 6 was 5.3% (range −13 to 24%), a statistically significant increase (*P*=0.0069 by paired *t*-test). Paradoxically, the only patient who had decreased left hip uptake at cycle 6 demonstrated a 10% increase in L4 uptake on the same scan compared to baseline imaging. SUVmean at L4 in the patient with cycle 3 imaging was unchanged compared to baseline.

DEXA was performed for the lumbar spine, L4, and the left hip at baseline, end of cycle 3 and end of cycle 6. There was no significant change in BMD at the lumbar spine or in the hip. However, at L4, there was progressive increase in BMD at each interval, with a significant increase of mean BMD of 6.1% at cycle 6 compared to baseline (*P* =0.009 by pairwise *t*-test). At L4, change in BMD measurements correlated significantly with NaF measurements (*r*=0.92, *P*=0.03) by Pearson’s test. In contrast, there was no significant correlation at the left hip or lumbar spine (*P*=0.58).

Dynamic NaF PET imaging of the thorax in a subset of three patients receiving DKN-01 showed a higher rate of accumulation at cycle 6 compared to baseline. This increase was statistically significant by analysis of variance (*P*<0.05). NaF accumulation was measured by drawing a threshold VOI encircling T10 as cortical background using an identical technique as for static imaging analysis. A VOI of at least 3 cm^3^ was also drawn in the left atrium as a blood pool surrogate. Mean time activity curves of blood pool and T10 aggregated across three patients are shown in Figure 1. A Patlak graphical plot was used to estimate the tumor influx constant (Ki).^10, 11^ A 24 min cutoff was used to fit the curve, as a steady state was achieved in <24 min for each individual subject.

In summary, static NaF PET imaging showed that the SUVmean of non-myelomatous cortical bone was stable to higher on treatment for all patients at L4 and for all except one patient at left hip. The rate of NaF accumulation at T10 by Patlak analysis was also higher on treatment for the subset of three patients who underwent dynamic NaF imaging.

While the level of increased NaF uptake observed in our study did not exceed the accepted 30% interscan variability threshold,^8^ the overall statistically significant and unidirectional change in NaF uptake among clinical responders (stable or improved disease), even among this small sample size, is supportive of an underlying alteration of regional fluoride accumulation. It is important to underscore that MM patients have accelerated bone loss compared to age-matched controls, exacerbated by the use of glucocorticoids as standard treatment. None of the enrolled patients were prescribed bisphosphonate therapy while on DKN-01 therapy, so even stable bone density and NaF uptake levels may imply some remediation of expected osteoporotic effects and suggests a possible role of targeting DKK1 as a bone-anabolic treatment. It is uncertain how longer duration of DKN-01 would impact global bone metabolism and NaF PET measurements.

Measurement of BMD at the lumbar spine and at the hip did not show a significant increase after six cycles. It is possible that this interval may be insufficient for an increase to be detectable by BMD. Interestingly, an increase in BMD at L4 was observed, and this also correlated with findings by NaF PET imaging.

Anti-DKK1 antibody has been shown in preclinical models to induce bone-anabolic activity and remediate osteoporotic bone. A phase IB trial of another anti-DKK1 antibody in relapsed/refractory MM, BHQ880 also showed excellent tolerability.^4^ In this trial, there was a general trend toward increased BMD over time, mainly after 12 cycles of treatment. Of note, in the BHQ880 trial, BHQ880 was given in combination with zoledronic acid. This limits the ability to determine the specific effect of BHQ880 on BMD.

We anticipate that as the anti-DKK1 antibody, DKN-01, and newer therapies are translated into patient care, there will be a need for noninvasive, robust and specific biomarkers to measure change in bone microenvironment. In this study, we provide preliminary results suggesting that quantitative NaF PET imaging may be a sensitive technique for measuring changes in bone quantity. Anatomic and functional data from multimodality imaging are not currently incorporated into the response criteria, but could potentially provide a means for simultaneously assessing tumor behavior as well as regional bone homeostasis.

## Figures and Tables

**Figure 1:**
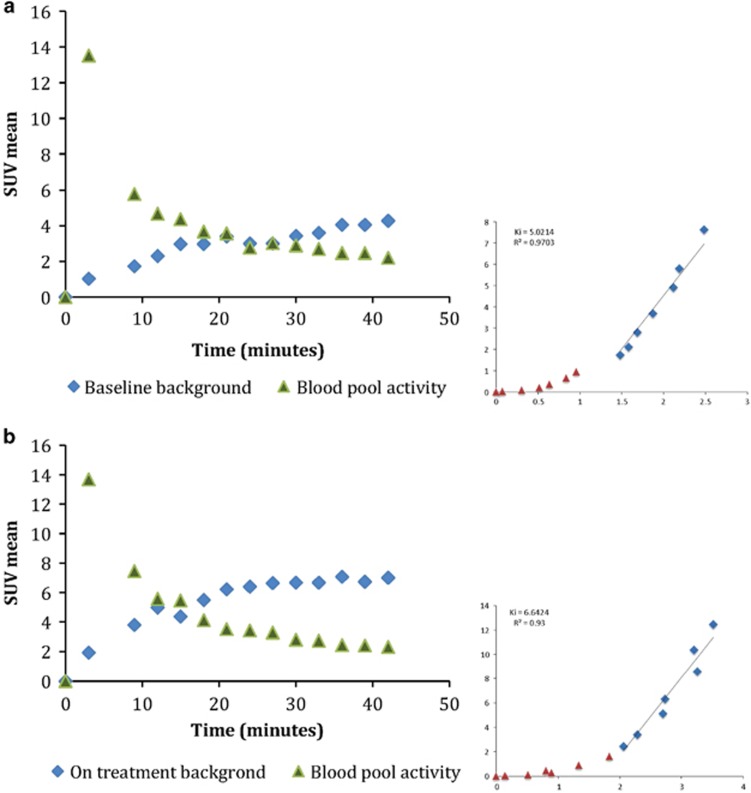
(**a**) Time activity curves of left atrial blood pool and cortical bone activity measured at T10 at baseline. Patlak plot with slope of the best fit line (Ki=5.0, *R*^2^=0.97). (**b**) Time activity curve of left atrial blood pool and cortical bone measured at T10 at cycle 6 and Patlak plot with slope of the best fit line (Ki=6.6, *R*^2^=0.93).

**Table 1:** Baseline characteristics

Age, median (range)	63 (53–80)
Time since diagnosis, median (range), years	3.9 (0.5–7.8)
	
*Sex,* n *(%)*	
Male	5 (62.5)
Female	3 (37.5)
	
*ISS stage*	
I	2 (25%)
II	4 (50%)
III	1 (12.5%)
Autologous stem cell transplant	4 (50%)
No. of prior therapies, median (range)	3 (2–5)

Abbreviation: ISS, International system staging. ISS stage for one subject not available.
